# The multi-omics analysis identifies a novel endoplasmic reticulum stress and immune related genes signature in lung adenocarcinoma

**DOI:** 10.1007/s12672-025-03033-w

**Published:** 2025-07-01

**Authors:** Danhe Huang, Yuying Liu, Mingyu Yuan, Xiongwei Wang, Lianqing Hong

**Affiliations:** Department of Pathology, Nanjing Integrated Traditional Chinese and Western Medicine Hospital, Nanjing, China

**Keywords:** Endoplasmic reticulum stress, Immune microenvironment, Prognostic, Lung adenocarcinoma, LASSO

## Abstract

**Background:**

Lung adenocarcinoma (LUAD), a prevalent and aggressive malignancy, necessitates improved prognostic tools and therapeutic insights. While endoplasmic reticulum stress (ERS) and tumor-immune interactions are recognized as key cancer hallmarks, their combined prognostic potential in LUAD remains insufficiently explored.

**Methods:**

Utilizing transcriptomic and clinical data from the TCGA-LUAD cohort, we developed an ERS-immune prognostic signature through Least Absolute Shrinkage and Selection Operator algorithm. A clinical nomogram integrating risk scores with established prognostic factors was established. Tumor microenvironment characteristics were evaluated using the CIBERSORT and ESTIMATE algorithm. The changes following the CR2 gene knockdown in NSCLC cells were evaluated through CCK-8 assay and Transwell assays.

**Results:**

The 10-gene signature effectively stratified patients into distinct risk groups with significant survival differences. The nomogram demonstrated enhanced predictive accuracy compared to traditional staging systems. High-risk patients exhibited immunosuppressive features. CR2 knockdown significantly reduced cellular proliferation and inhibited metastatic capacity.

**Conclusion:**

This integrated ERS-immune signature provides clinically relevant prognostic stratification and reveals potential therapeutic vulnerabilities in LUAD, offering a framework for personalized treatment strategies.

**Supplementary Information:**

The online version contains supplementary material available at 10.1007/s12672-025-03033-w.

## Introduction

Lung adenocarcinoma (LUAD), the most prevalent histological subtype of non-small cell lung cancer (NSCLC), remains a major cause of cancer-related mortality worldwide [1, 2]. Despite advancements in targeted therapy and immunotherapy, the prognosis for LUAD patients remains poor due to tumor heterogeneity and resistance mechanisms [3, 4]. Current prognostic models primarily rely on clinical and pathological parameters, which lack sufficient precision in capturing the complex molecular landscape of LUAD. Therefore, novel biomarkers and integrative prognostic models are urgently needed to improve risk stratification and therapeutic decision-making.

Endoplasmic reticulum stress (ERS), triggered by protein misfolding and accumulation in the ER lumen, plays a crucial role in tumorigenesis by modulating cell survival, apoptosis, and metabolic reprogramming [5–8]. When ER homeostasis is disrupted, cells activate the unfolded protein response (UPR), an adaptive mechanism aimed at restoring normal protein folding and mitigating cellular stress [9]. However, chronic activation of the UPR in cancer cells not only enhances their survival under adverse conditions but also contributes to drug resistance and immune evasion [10, 11]. In LUAD, persistent ERS can drive tumor progression by promoting epithelial-mesenchymal transition [12], enhancing angiogenesis, and reprogramming metabolic pathways for rapid proliferation [13]. Furthermore, ERS has been implicated in modulating autophagy and ferroptosis, processes that influence tumor cell fate and therapeutic responses [14]. Emerging evidence suggests that ERS is intricately linked to tumor immunity, shaping the tumor immune microenvironment through multiple mechanisms [15]. ERS can suppress antigen presentation by downregulating major histocompatibility complex molecules, thereby impairing immune recognition and facilitating immune escape [16]. Additionally, it influences the polarization and function of tumor-infiltrating immune cells, such as macrophages, dendritic cells, and T cells [17]. For instance, ERS-induced activation of the PERK-eIF2α-CHOP pathway can drive an immunosuppressive phenotype in tumor-associated macrophages [18], while XBP1 signaling has been shown to promote the exhaustion of cytotoxic T lymphocytes. These alterations in immune cell function contribute to an immunosuppressive microenvironment, further enabling LUAD progression and therapy resistance [19]. Despite these insights, the combined prognostic value of ERS-related and immune-related genes in LUAD remains largely unexplored. Understanding how ERS shapes the immune landscape in LUAD is essential for identifying novel therapeutic targets and developing integrative prognostic models to refine risk stratification.

In this study, we conducted a comprehensive multi-omics analysis to establish an ERS-immune prognostic signature for LUAD. Our findings revealed distinct immune microenvironment characteristics between risk groups, shedding light on immunosuppressive mechanisms and potential therapeutic vulnerabilities.

## Materials and methods

### Data collection and cohort processing

Clinical-pathological records and RNA-seq profiles of lung adenocarcinoma (LUAD) cohorts were sourced from the TCGA database. Complementary expression matrices (GSE31210, GSE72094) with annotated clinical metadata were accessed via GEO platform. Raw sequencing files underwent quality-controlled preprocessing, including TPM normalization for cross-sample comparability and exclusion of cases with missing survival documentation. Demographic parameters (age, gender), disease staging (TNM classification), and prognostic indicators were systematically curated. Independent GEO cohorts were subsequently employed for validation analyses.

### Weighted correlation network analysis

Transcriptome data from TCGA-LUAD were analyzed using WGCNA to construct gene co-expression networks. Outliers were removed, and a soft thresholding power (β chosen to achieve scale-free topology fit R² > 0.90) was selected to ensure scale-free topology. Modules were identified with a minimum size of 30 using dynamic tree-cutting, visualized in a dendrogram with color coding. Key modules correlated with immune scores were highlighted in a heatmap. Genes from these modules were classified as endoplasmic reticulum stress-related immune genes.

### Differentially expressed genes and functional enrichment analysis

To identify differentially expressed genes (DEGs) associated with ERS and immune genes. We performed tumor-normal comparisons which revealed significant expression variations analyzed through limma’s empirical Bayes framework, applying implementing a dual threshold of minimum 2-fold expression difference (|log2FC| > 1) and false discovery rate-adjusted significance (FDR < 0.05). To explore the biological significance of the identified DEGs, Gene Ontology (GO) and Kyoto Encyclopedia of Genes and Genomes (KEGG) pathway enrichment analyses were conducted using the “clusterProfiler” package in R.

### Construction and validation of the predictive model

DEGs linked to ERS and immune-related genes were analyzed. A predictive algorithm was constructed using Least Absolute Shrinkage and Selection Operator (LASSO) regression applied to molecular profiles from the TCGA-LUAD dataset. Biomarker selection was performed through regularization path optimization, with retained variables determined by the penalty parameter (λ) that minimized cross-validation error. Individual prognostic risk scores were computed as the dot product of gene expression vectors and their corresponding regression coefficients. The robustness of the model was further validated in independent GEO cohorts.

### Establishment of a nomogram

A clinical nomogram was developed to integrate the risk score with traditional clinical factors such as age, gender, and TNM stage. The performance of the nomogram was evaluated using calibration curves.

### Estimation of the tumor microenvironment

The immune landscape of LUAD patients was analyzed using the CIBERSORT algorithm to estimate the relative abundance of 28 immune cell types. Additionally, the ESTIMATE algorithm was used to calculate immune and stromal scores, providing an assessment of TME heterogeneity between risk groups.

### Tumor mutational burden analysis

Somatic mutation data from TCGA-LUAD patients were analyzed using the “maftools” package. Tumor mutational burden (TMB) was calculated as the total number of nonsynonymous mutations per megabase. The relationship between TMB and the risk score was assessed, and survival differences between high- and low-TMB groups were evaluated using Kaplan-Meier analysis.

### Drug sensitivity analysis

To explore the therapeutic implications of the ERS-immune signature, the half-maximal inhibitory concentration (IC50) of common chemotherapeutic agents and targeted therapies was predicted using the “pRRophetic” package. Differential drug responses between risk groups were compared using Wilcoxon rank-sum tests.

### Cell culture and transfection

LUAD cell lines A549 and H1299 were acquired from the American Type Culture Collection (ATCC, Virginia, USA). These cell lines were maintained in Dulbecco’s Modified Eagle Medium (DMEM; Biological Industries, Israel) containing 10% fetal bovine serum (FBS; Gibco, Thermo Fisher Scientific, USA) under standard culture conditions. For genetic manipulation experiments, we employed lipid-mediated transfection methodology. Specifically, gene-specific small interfering RNA (siRNA) targeting CR2 and corresponding negative control siRNA (Ribobio, Guangzhou, China) were introduced into cells using Lipofectamine 2000 transfection reagent (Thermo Fisher Scientific, USA), following the established protocols provided by the manufacturer. Post-transfection, cells were harvested for subsequent functional characterization studies.

### CCK-8

Cellular viability was quantified via CCK-8 assays (Beyotime, Guangzhou, China). Post-transfection cells (5 × 10³/well) seeded in 96-well plates were analyzed at 24-, 48-, and 72-hour intervals through 450 nm absorbance readings after incubation with the reagent. Triplicate measurements ensured reproducibility.

### Migration and invasion assessment

Cell migration and invasion potential were assessed using Matrigel-coated Transwell chambers (#3422, Corning, USA) to evaluate invasive potential. For invasion assays, serum-starved cell suspensions (1 × 10⁵) were loaded into the upper compartments, with chemoattractant-containing medium in the lower chamber. After 24 h, cells that had invaded to the lower chamber were stained with crystal violet and enabled microscopic quantification (200× magnification). For migration assay, the same method was used but with chambers containing an uncoated membrane.

### Statistical analysis

All statistical analyses were performed using R (version 4.2.0) and GraphPad Prism. Continuous variables were compared using Student’s t-test or Wilcoxon rank-sum test. Survival analysis was conducted using Kaplan-Meier curves and Cox proportional hazards regression. A two-tailed P value of < 0.05 was considered statistically significant.

## Results

### Molecular characterization of ERS-immune axis

**Fig. 1:**
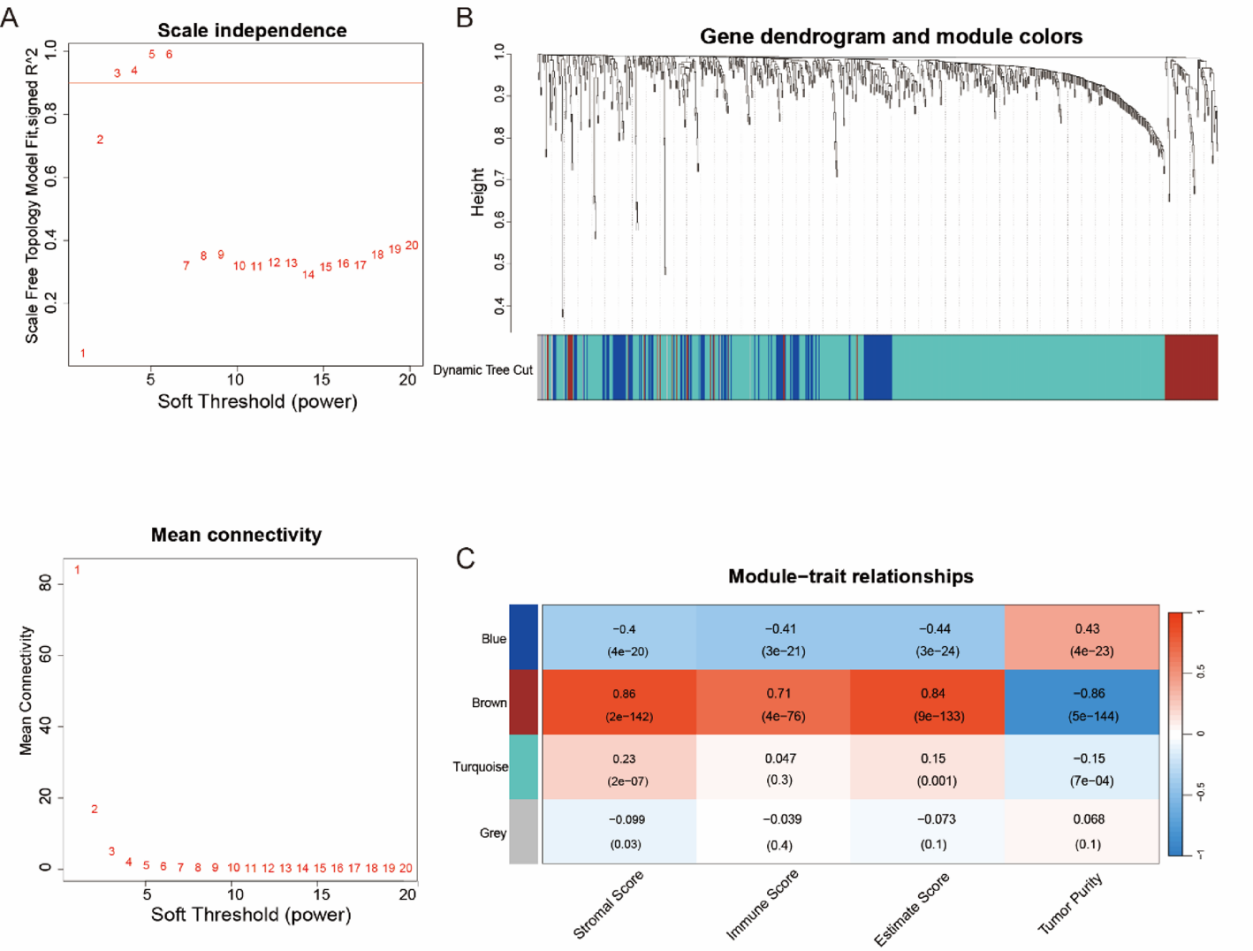
Co-expression network structure. (A) Connectivity parameter optimization for scale-free architecture validation through soft threshold power evaluation. (B) Hierarchical clustering dendrogram with color-coded modules reflecting topological dissimilarity. (C) Module-trait association matrix with color gradients (red: high; blue: low correlation coefficients) and statistical significance indicated parenthetically

Stromal and immune microenvironment metrics were quantified using ESTIMATE algorithm in TCGA-LUAD cohort. WGCNA identified stress-responsive immune modules, optimized through β = 3 soft-threshold selection (Fig. 1A). Chromatically defined modules revealed brown cluster showing strongest immune score correlation (Cor = 0.71; *P* = 4 × 10^− 76^; Fig. 1B, C), yielding 784 hub genes designated ERS-immune genes.

### Functional enrichment analysis

**Fig. 2:**
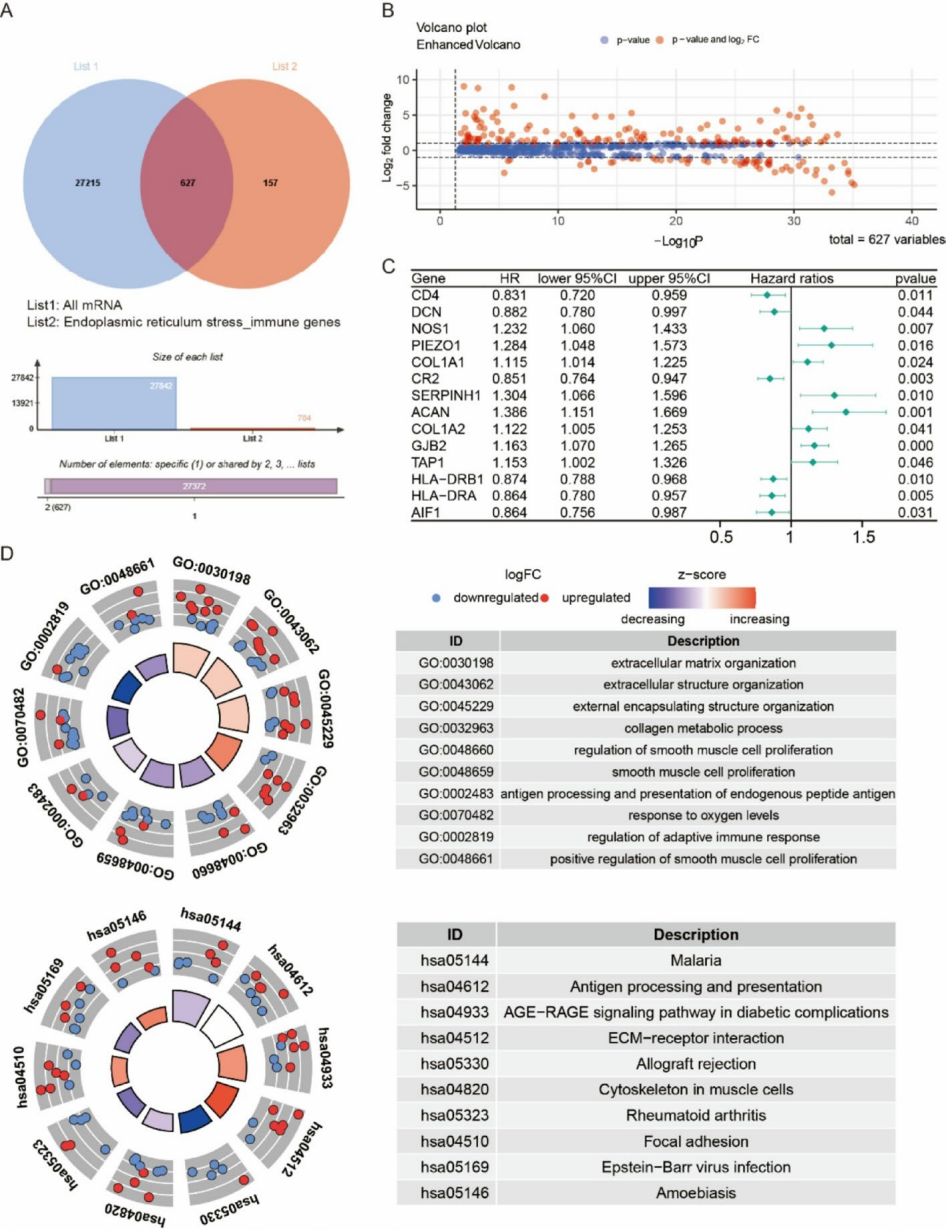
Functional enrichment analysis. (A) Venn diagram showing the overlap between all mRNA (List 1) and endoplasmic reticulum stress and immune genes (List 2). (B) Volcano plot displaying the DEGs. (C) Forest plot showing the hazard ratios (HR), 95% confidence intervals, and P-values for genes associated with prognosis. (D) Circular visualization of GO and KEGG pathway enrichment analysis

After further intersected with 784 hub genes and total mRNA, 628 genes were obtained (Fig. 2A). Volcano visualization confirmed tumor-normal ERS-immune genes differential expression (Fig. 2B). Survival-associated candidates emerged through univariate Cox screening (Fig. 2C). Gene Ontology Functional enrichment analysis revealed that the differentially expressed genes (DEGs) were significantly enriched in extracellular matrix restructuring, KEGG pathway analysis further highlighted key enrichments in malaria, antigen presentation, AGE-RAGE signaling (Fig. 2D).

### Predictive model development

**Fig. 3:**
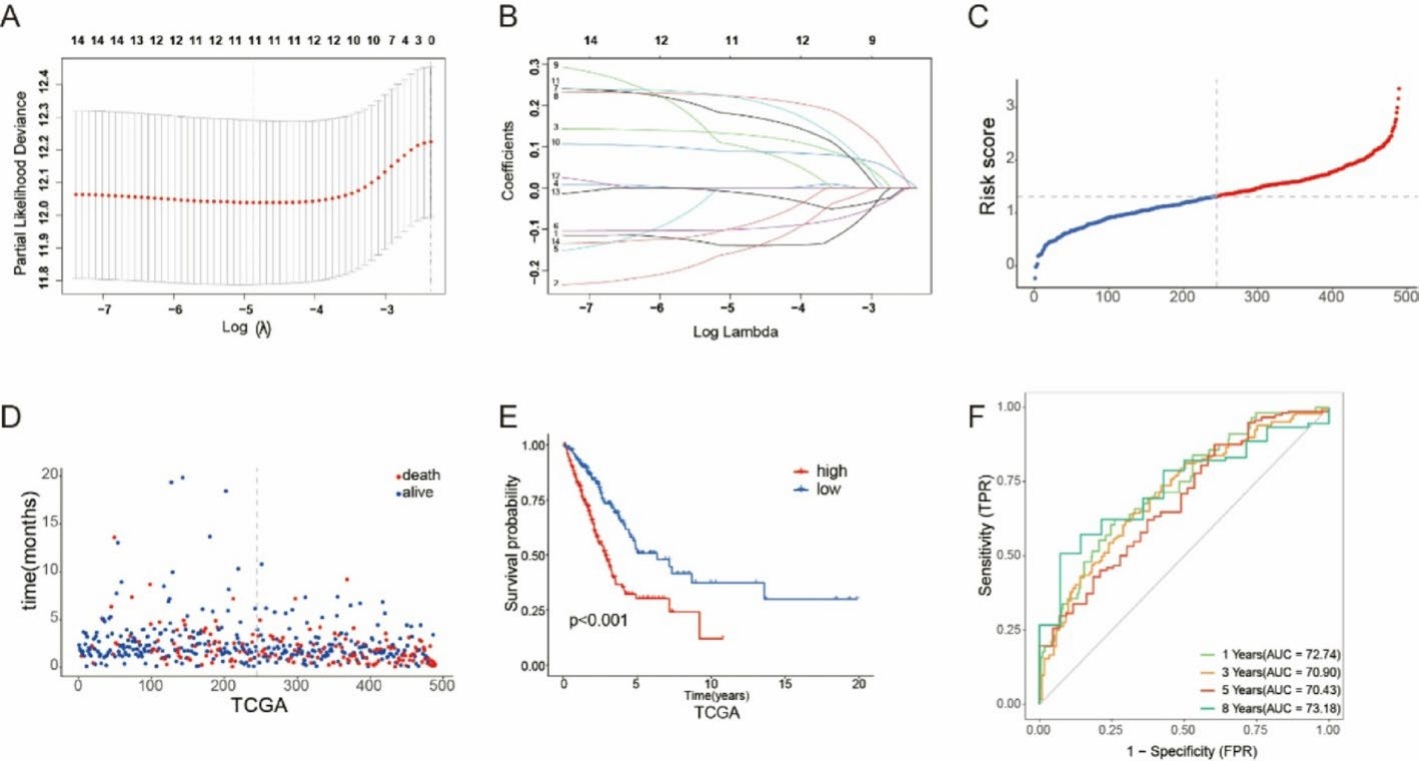
Construction of the sisk score model. (A) Coefficient profile plot of selected genes. (B) Lasso Cox regression with 10-fold cross-validation for parameter tuning. (C, D) Risk score distribution and survival status of patients in TCGA. (E) Kaplan-Meier survival analysis comparing high- and low-risk groups. (F) ROC curve evaluating the prognostic accuracy in TCGA

The LASSO regression method was employed to construct a prognostic model using the TCGA-LUAD cohort. This analysis identified a 10-gene signature (CD4, DCN, HLA-DRA, AIF1, CR2, TAP1, NOS1, COL1A2, GJB2, ACAN and SERPINH1) significantly associated with LUAD patient outcomes (Fig. 3A, B). Prognostic indices stratified patients into distinct survival cohorts (Fig. 3C, D), with high-risk group showing significant mortality elevation (Fig. 3E). Moreover, ROC curve analysis demonstrated the robust prognostic performance of the model, with AUC values of 72.74, 70.90, 70.43, and 73.18 for 1-, 3-, 5-, and 8-year survival, respectively, underscoring its potential clinical utility in prognostic prediction (Fig. 3F). To further validate the robustness of model, we performed independent validation using the GSE72094 and GSE31210 datasets, which yielded consistent and clinically promising results (Figure S1).

### Construction of a prognostic nomogram

**Fig. 4:**
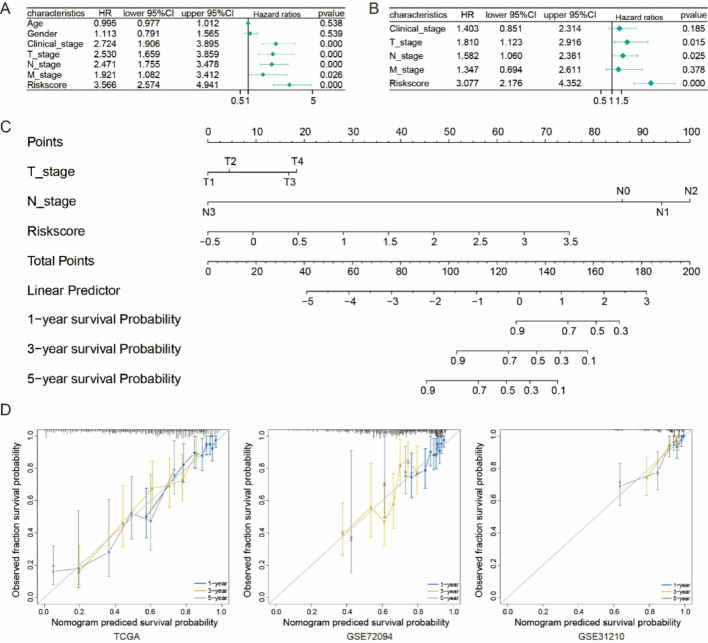
Nomogram construction. (A) Forest plot showing univariate Cox analysis of clinical features (age, gender, clinical stage, T stage, N stage, M stage) and risk score. (B) Forest plot showing multivariate Cox analysis of clinical features (clinical stage, T stage, N stage, M stage) and risk score. (C) Nomogram based on clinical features and risk score. (D) Calibration curves for the TCGA, GSE27094, and GSE31210 datasets

Univariate and multivariate Cox regression analyses identified risk scores and T/N stage as independent prognostic factors for LUAD (Fig. 4A, B). A refined nomogram incorporating these factors was developed (Fig. 4C). Calibration curves demonstrated its strong predictive performance for 1-, 3-, and 5-year OS in the TCGA. These results were further confirmed using GSE72094 and GSE312100, reinforcing the stability of the model and clinical relevance (Fig. 4D).

### Tumor microenvironment analysis

**Fig. 5:**
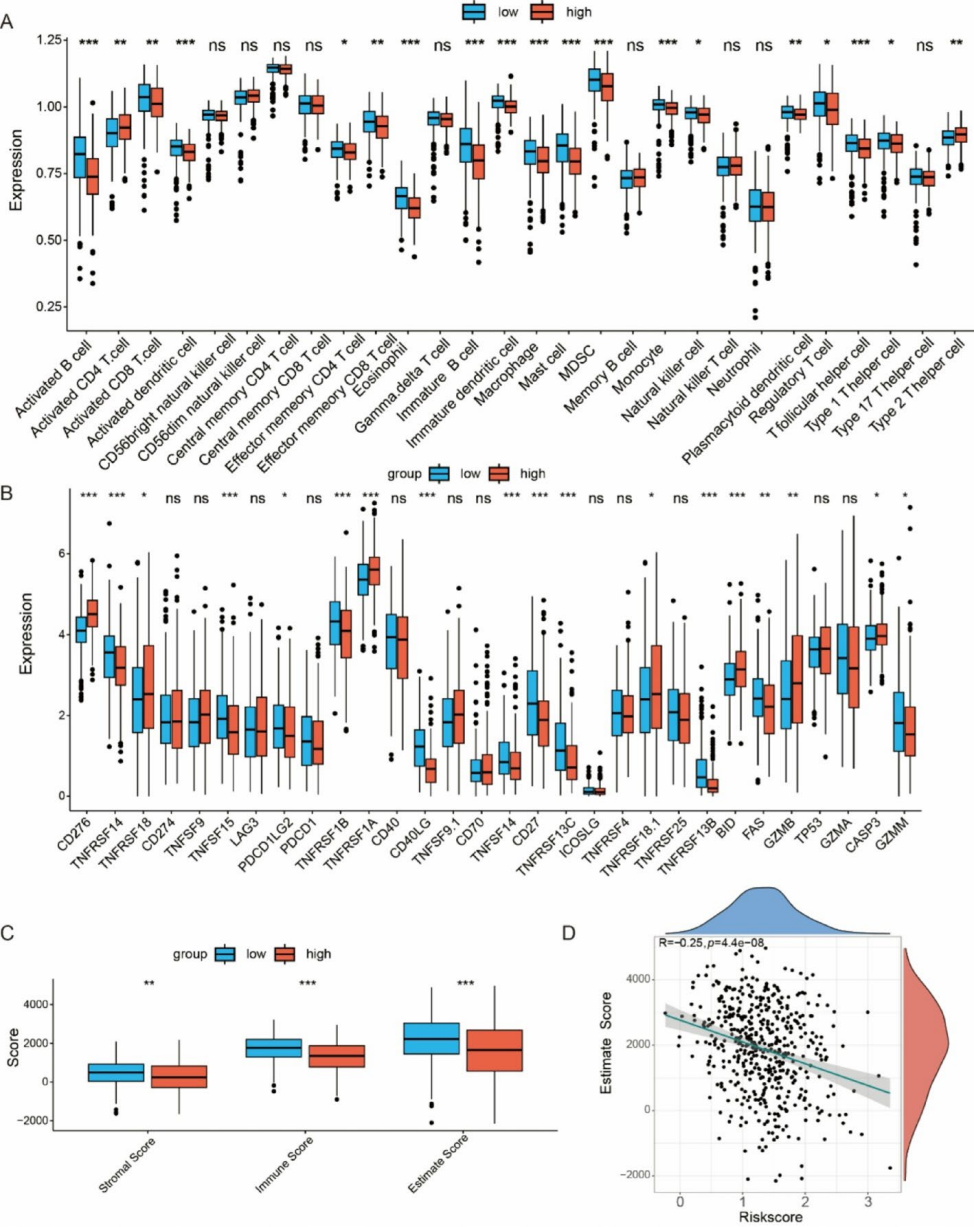
Immune infiltration analysis between risk groups. (A) Distribution of 28 immune cell subsets between high and low-risk groups. (B) Boxplot showing the expression of immune checkpoint molecules, inflammatory factors, and the cytolytic molecule gene family in the high-risk and low-risk groups. (C) Boxplot displaying the expression levels of immune scores. (D) Negative correlation between ESTIMATE scores and risk score. *p < 0.05, ** *p* < 0.01, *** *p* < 0.001

The tumor microenvironment was characterized using CIBERSORT and ESTIMATE algorithm to assess immune cell infiltration. The high-risk group exhibited significantly lower immune cell infiltration, suggesting an immunosuppressive phenotype (Fig. 5A). Immune checkpoint molecules, inflammatory factors, and the cytolytic molecule gene family analysis revealed lower expression of TNFRSF14, TNFRSF15, PDCD1LG2, CD40LG, TNFSF14, CD27, TNFRSF13C, TNFRSF13B, FAS and GZMM in high-risk patients (Fig. 5B). Furthermore, ESTIMATE analysis indicated significantly lower immune, stromal, and ESTIMATE scores in the high-risk group (Fig. 5C). Further correlation analysis revealed a negative association between ESTIMATE scores and risk score (Fig. 5D), suggesting a potential resistance to immunotherapy and an immunosuppressive TME.

### Tumor mutational burden analysis

**Fig. 6:**
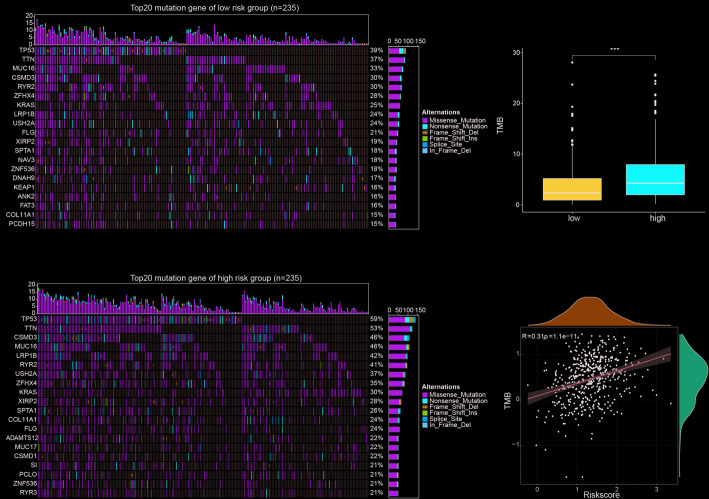
Correlation of prognostic model with tumor mutation burden. (A, B) Waterfall plots showing the distribution differences of the top 20 mutated genes in the low-risk group (A) and high-risk group (B). (C) Boxplot showing the distribution of TMB in the high-risk and low-risk groups. (D) Scatter plot showing the correlation between risk score and TMB. *p < 0.05, **p < 0.01, ***p < 0.001

To further investigate genomic differences between risk groups, we analyzed TMB in the TCGA cohort. The top five most frequently mutated genes in the low-risk group were TP53, MUC16, CSMD3, RYR2, and ZFHX4 (Fig. 6A), whereas the high-risk group exhibited frequent mutations in TP53, TTN, CSMD3, MUC16, and LRP1B (Fig. 6B). Notably, the high-risk group displayed significantly higher tumor mutational burden (Fig. 6C), and correlation analysis confirmed a positive association between TMB and the risk score (Fig. 6D).

### Drug sensitivity analysis

**Fig. 7:**
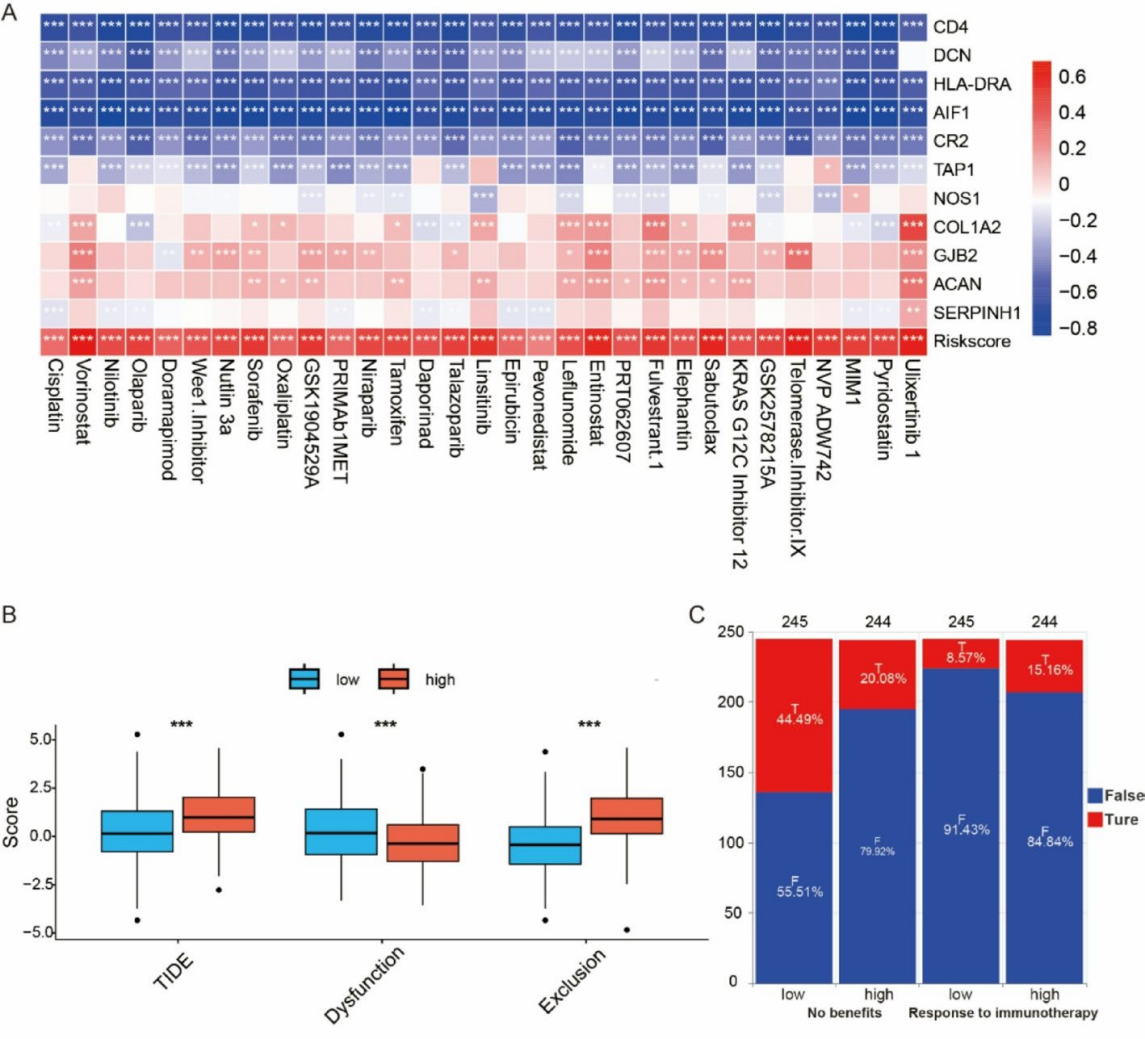
Association of drug sensitivity, immune response, and risk score. (A) Correlation heatmap of gene expression and therapeutic drug responses (chemotherapy and targeted therapies). (B) TIDE score comparison between high- and low-risk groups. (C) Immunotherapy response rates stratified by risk group. *p < 0.05, **p < 0.01, ***p < 0.001

To explore therapeutic implications, we assessed drug sensitivity using the “pRRophetic” package. Correlation analysis revealed a strong association between risk scores and drug sensitivity (Fig. 7A). Further evaluation using TIDE and Exclusion scores indicated higher scores in the high-risk group, suggesting potential immune evasion. Conversely, a lower Dysfunction score was observed in the high-risk group, indicating impaired immune function (Fig. 7B). In the prediction of immunotherapy response, a greater proportion of individuals in the high-risk group did not benefit from immunotherapy, while patients in the low-risk group showed a better response to treatment compared to those in the high-risk group (Fig. 7C). Therefore, we believe that the risk score-based grouping has predictive and guiding value for immunotherapy outcomes.

**Fig. 8:**
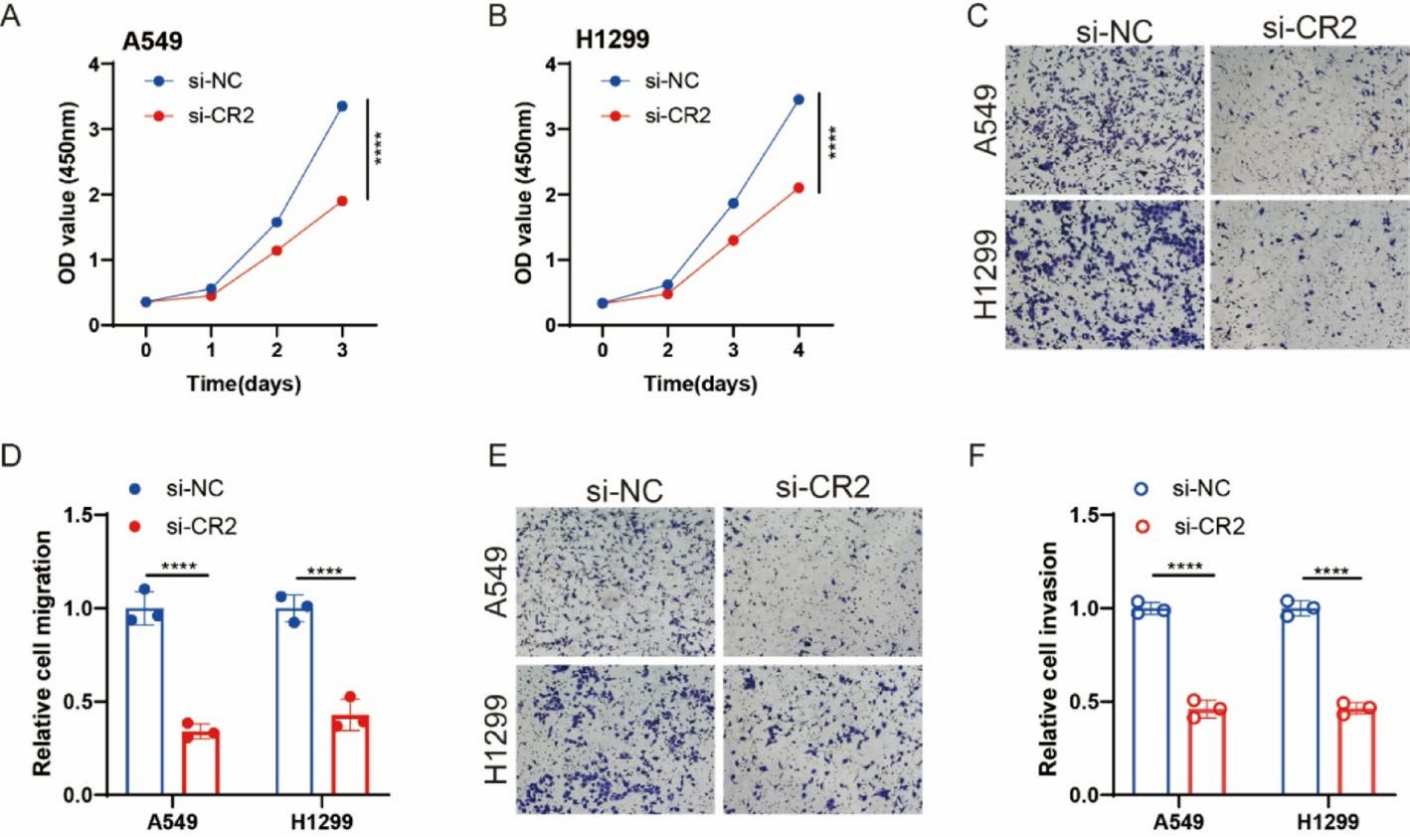
Functional characterization of CR2 in LUAD cells. (A, B) CR2 knockdown significantly reduced cell viability assessed by CCK-8 assay in A549 and H1299 cells. (C, D) CR2 silencing markedly impaired cell migration capacity in Transwell assays (uncoated membrane) using A549 and H1299 cells. (E, F) CR2 depletion potently suppressed cell invasion capacity in Transwell assays (Matrigel-coated membrane) using A549 and H1299 cells. Data represent mean ± SD; ****P < 0.0001

### Knowdown CR2 inhibits proliferation and metastasis of LUAD Cells

Through drug sensitivity analysis, we identified CR2 as a gene closely associated with LUAD treatment. Given its limited prior research in lung cancer, we hypothesize that CR2 may serve as a novel therapeutic target. Functional validation experiments showed that CR2 knockdown significantly inhibited LUAD cell proliferation and reduced metastasis (Fig. 8). These findings suggest that CR2 may be a promising target for LUAD therapy, further supporting the clinical relevance of our ERS-immune signature.

## Discussion

In this study, we systematically explored the relationship between ERS, immune-related genes, and their prognostic significance in LUAD. Using the ESTIMATE algorithm and WGCNA, we identified a set of ERS-immune genes that exhibited significant prognostic value. Functional enrichment analyses revealed that these genes were primarily involved in extracellular matrix organization, malaria, antigen processing and presentation, and the AGE-RAGE signaling pathway, suggesting their critical roles in tumor progression and immune regulation.

To further assess their prognostic relevance, we constructed a risk model using LASSO Cox regression analysis, identifying a 10-gene signature that effectively stratified LUAD patients into high- and low-risk groups. GJB2 is associated with glycolysis and immunosuppression in human tumors. Studies have shown that HDLBP-mediated GJB2 RNA stabilization enhances glycolysis and CD8 T cell exhaustion in the progression of LUAD [20]. ACAN can serve as a novel basement membrane-related gene to predict prognosis in LUAD [21]. AIF-1 may promote the aggressive behavior of NSCLC by activating p38-MAPK and JAK/STAT signaling [22]. Kaplan-Meier survival analysis demonstrated that high-risk patients had significantly worse OS, and the model exhibited strong predictive accuracy with AUC values exceeding 0.70 across multiple time points. External validation in GEO datasets confirmed the robustness and clinical utility of this model. Compared to existing approaches, this model demonstrates comprehensive performance improvements in time-dependent ROC analysis. Specifically, our model achieved AUC values of 0.729, 0.709, and 0.704 at 1-, 3-, and 5-year prediction time points, respectively. These results represent significant improvements over the ferroptosis features in tumor-associated macrophages model (0.628, 0.629, 0.588) [23] and the brain metastasis-related model (0.634, 0.679, 0.615) [24]. Notably, our model exhibited superior stability with increasing prediction time spans, maintaining an AUC above 0.7 even at the 5-year mark. This robust performance underscores the reliability of model for long-term prognostic prediction and provides clinicians with a more accurate tool for extended outcome assessment. While our signature demonstrates superior prognostic performance, its clinical implementation requires cost-effectiveness evaluation compared to standard NCCN guideline biomarkers. The potential added value must be weighed against the additional expenses of genomic profiling. However, the improved risk stratification accuracy may ultimately reduce costs by avoiding overtreatment in low-risk patients, and enabling earlier, more targeted interventions for high-risk cases. Future health-economic studies should formally compare long-term outcomes versus resource utilization between these approaches.

Beyond prognosis, we integrated the risk score into a comprehensive nomogram that combined clinical parameters such as TNM stage and patient demographics. This nomogram outperformed traditional staging systems in predicting patient survival, as demonstrated by calibration curves, emphasizing its potential as a valuable clinical tool. Further investigation of the TME revealed that the high-risk group exhibited reduced immune cell infiltration, suggesting an immunosuppressive phenotype that may contribute to immune evasion and resistance to immunotherapy. Additionally, TMB analysis revealed distinct mutation patterns between the high- and low-risk groups, with high-risk patients exhibiting elevated TMB, a factor known to influence response to immunotherapy.

Drug sensitivity analysis identified a strong correlation between the risk score and drug response patterns, highlighting potential therapeutic vulnerabilities. Notably, CR2 was identified as a novel therapeutic target, with functional validation experiments confirming that its knockdown significantly inhibited LUAD cell proliferation and migration. These findings suggest that CR2 may serve as a promising treatment target in LUAD.

## Conclusions

In summary, we identified and validated a novel ERS-IRGs signature with significant prognostic and therapeutic implications in LUAD. Our risk model effectively stratifies patients by survival outcomes and immune landscape, providing a clinically relevant tool for treatment decision-making. Furthermore, our findings suggest that targeting CR2 may offer a novel therapeutic approach for LUAD patients. Future studies are warranted to explore the underlying mechanisms of ERS-immune interactions and validate CR2 as a potential drug target in LUAD.

## Electronic supplementary material

Below is the link to the electronic supplementary material.

Supplementary Material 1

## Data Availability

Publicly available datasets were analyzed in this study.

## References

[1] Siegel RL, Miller KD, Wagle NS, Jemal A. Cancer statistics, 2023. CA Cancer J Clin. 2023;73(1):17–48. 36633525 10.3322/caac.21763

[2] Nasim F, Sabath BF, Eapen GA. Lung Cancer. Med Clin North Am. 2019;103(3):463–73. 30955514 10.1016/j.mcna.2018.12.006

[3] Wu F, Fan J, He Y, Xiong A, Yu J, Li Y, Zhang Y, Zhao W, Zhou F, Li W, et al. Single-cell profiling of tumor heterogeneity and the microenvironment in advanced non-small cell lung cancer. Nat Commun. 2021;12(1):2540. 33953163 10.1038/s41467-021-22801-0 PMC8100173

[4] de Sousa VML, Carvalho L. Heterogeneity in lung Cancer. Pathobiology. 2018;85(1–2):96–107. 29635240 10.1159/000487440

[5] Chen X, Cubillos-Ruiz JR. Endoplasmic reticulum stress signals in the tumour and its microenvironment. Nat Rev Cancer. 2021;21(2):71–88. 33214692 10.1038/s41568-020-00312-2 PMC7927882

[6] Anerillas C, Mazan-Mamczarz K, Herman AB, Munk R, Lam KG, Calvo-Rubio M, Garrido A, Tsitsipatis D, Martindale JL, Altes G, et al. The YAP-TEAD complex promotes senescent cell survival by Lowering Endoplasmic reticulum stress. Nat Aging. 2023;3(10):1237–50. 37667102 10.1038/s43587-023-00480-4 PMC11369890

[7] Fernandez A, Ordonez R, Reiter RJ, Gonzalez-Gallego J, Mauriz JL. Melatonin and Endoplasmic reticulum stress: relation to autophagy and apoptosis. J Pineal Res. 2015;59(3):292–307. 26201382 10.1111/jpi.12264

[8] Hu C, Xin Z, Sun X, Hu Y, Zhang C, Yan R, Wang Y, Lu M, Huang J, Du X, et al. Activation of ACLY by sect. 63 deploys metabolic reprogramming to facilitate hepatocellular carcinoma metastasis upon Endoplasmic reticulum stress. J Exp Clin Cancer Res. 2023;42(1):108. 37122003 10.1186/s13046-023-02656-7 PMC10150531

[9] Chen X, Shi C, He M, Xiong S, Xia X. Endoplasmic reticulum stress: molecular mechanism and therapeutic targets. Signal Transduct Target Ther. 2023;8(1):352. 37709773 10.1038/s41392-023-01570-w PMC10502142

[10] Khaled J, Kopsida M, Lennernas H, Heindryckx F. Drug resistance and Endoplasmic reticulum stress in hepatocellular carcinoma. Cells 2022, 11(4). 10.3390/cells11040632 PMC8870354 35203283

[11] Lou X, Gao D, Yang L, Wang Y, Hou Y. Endoplasmic reticulum stress mediates the myeloid-derived immune suppression associated with cancer and infectious disease. J Transl Med. 2023;21(1):1. 36593497 10.1186/s12967-022-03835-4 PMC9809056

[12] Yu M, Chen F, Wang H, Fu Q, Yan L, Chen Z, Li H, Jia M, Yang D, Hua X, et al. Endoplasmic reticulum stress mediates nickel chloride-induced epithelial–mesenchymal transition and migration of human lung cancer A549 cells through Smad2/3 and p38 MAPK activation. Ecotoxicol Environ Saf. 2023;249:114398. 36508813 10.1016/j.ecoenv.2022.114398

[13] Marciniak SJ. Endoplasmic reticulum stress in lung disease. Eur Respir Rev 2017, 26(144). 10.1183/16000617.0018-2017 PMC9488656 28659504

[14] Qi Z, Chen L. Endoplasmic reticulum stress and autophagy. Adv Exp Med Biol. 2019;1206:167–77. 31776985 10.1007/978-981-15-0602-4_8

[15] Bettigole SE, Glimcher LH. Endoplasmic reticulum stress in immunity. Annu Rev Immunol. 2015;33:107–38. 25493331 10.1146/annurev-immunol-032414-112116

[16] Thoma A, Earl KE, Goljanek-Whysall K, Lightfoot AP. Major histocompatibility complex I-induced Endoplasmic reticulum stress mediates the secretion of pro-inflammatory muscle-derived cytokines. J Cell Mol Med. 2022;26(24):6032–41. 36426551 10.1111/jcmm.17621 PMC9753450

[17] Ma X, Bi E, Lu Y, Su P, Huang C, Liu L, Wang Q, Yang M, Kalady MF, Qian J, et al. Cholesterol induces CD8(+) T cell exhaustion in the tumor microenvironment. Cell Metab. 2019;30(1):143–e156145. 31031094 10.1016/j.cmet.2019.04.002 PMC7061417

[18] Kolodeeva OE, Kolodeeva OE, Averinskaya DA, Makarova YA. Induction of the PERK-eIF2alpha-ATF4 pathway in M1 macrophages under Endoplasmic reticulum stress. Dokl Biochem Biophys. 2024;517(1):264–8. 39002013 10.1134/S1607672924600301 PMC11263228

[19] Zhu C, Xie Y, Li Q, Zhang Z, Chen J, Zhang K, Xia X, Yu D, Chen D, Yu Z, et al. CPSF6-mediated XBP1 3’UTR shortening attenuates cisplatin-induced ER stress and elevates chemo-resistance in lung adenocarcinoma. Drug Resist Updat. 2023;68:100933. 36821972 10.1016/j.drup.2023.100933

[20] Xu L, Zhou B, Jin K, Ge T, Deng M, Ding H, Xu X. HDLBP promotes Glycolysis and CD8(+) T cell exhaustion in lung adenocarcinoma by stabilizing GJB2 RNA. Am J Respir Cell Mol Biol 2025. 10.1165/rcmb.2024-0648OC 40343852

[21] Zhu X, Liu X, Qiu X, Niu Z, Dong W, Song Y. Prognostic roles of a novel basement membranes-related gene signature in lung adenocarcinoma. Front Genet. 2023;14:1100560. 36845403 10.3389/fgene.2023.1100560 PMC9946986

[22] Wang L, Zhao X, Zheng H, Zhu C, Liu Y. AIF-1, a potential biomarker of aggressive tumor behavior in patients with non-small cell lung cancer. PLoS ONE. 2022;17(12):e0279211. 36520870 10.1371/journal.pone.0279211 PMC9754194

[23] Ji T, Jiang J, Wang X, Yang K, Wang S, Pan B. Single-cell transcriptomics and machine learning unveil ferroptosis features in tumor-associated macrophages: prognostic model and therapeutic strategies for lung adenocarcinoma. Front Pharmacol. 2025;16:1598756. 40421217 10.3389/fphar.2025.1598756 PMC12104069

[24] Gong Z, Yu F, Li C, Zhao B, Wen M, Zhang S, Xu Z, Wu A, Zang R, Li Y et al. Four-gene prognostic signature and risk of brain metastasis of lung adenocarcinoma. Mol Carcinog 2025. 10.1002/mc.23922 PMC12183640 40222041

