# Supplementary material for: The multi-omics analysis identifies a novel endoplasmic reticulum stress and immune related genes signature in lung adenocarcinoma
Source: Discov Oncol. 2025 Jul 1;16:1190. doi: 10.1007/s12672-025-03033-w (PMC12214066; doi:10.1007/s12672-025-03033-w)
Supplement: Supplementary file 1 — Supplementary Material 1 [file 12672_2025_3033_MOESM1_ESM.docx]

Supplementary Figures


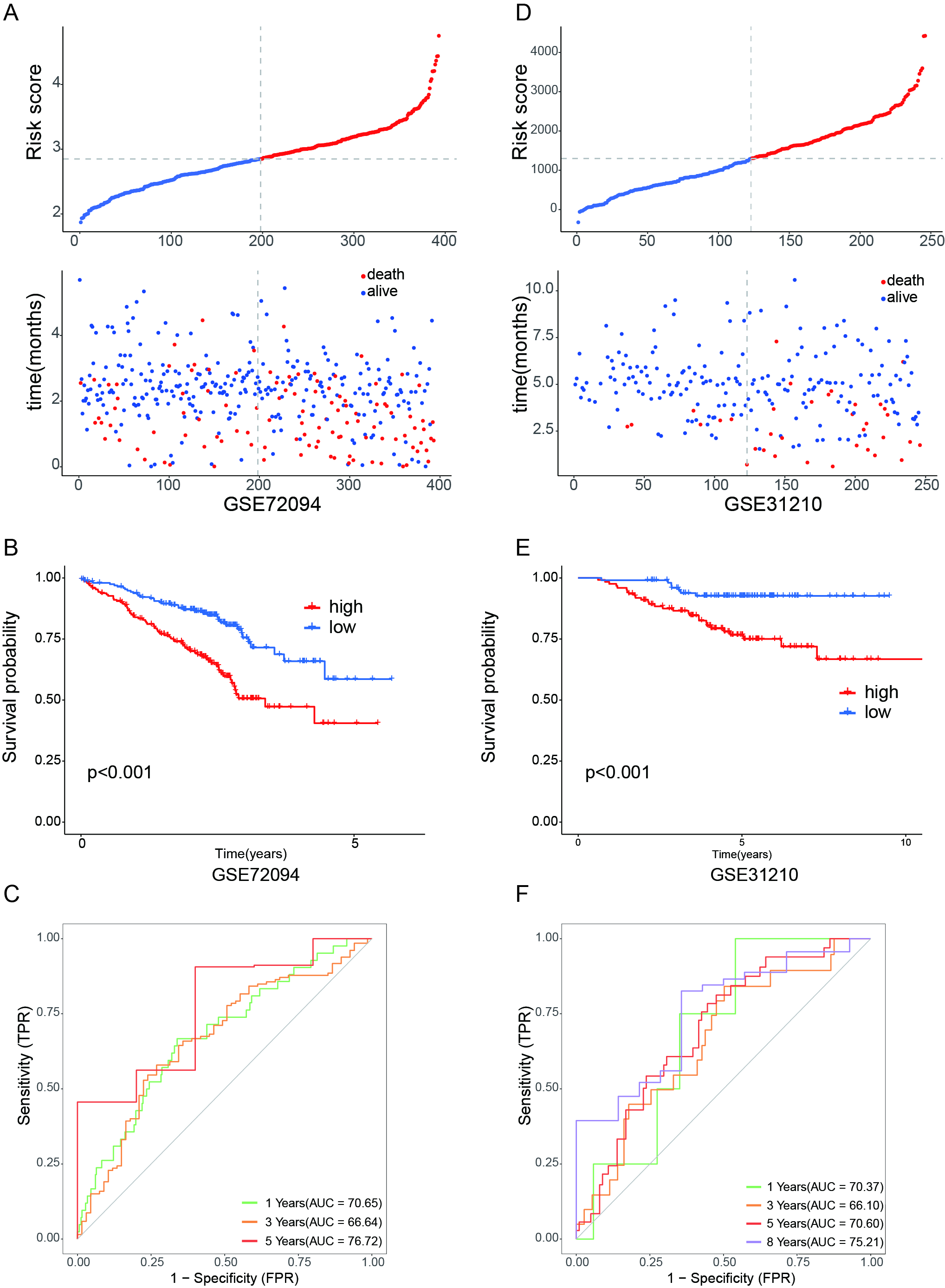


Figure S1. Validation of the sisk score model in independent cohorts. (A) Risk score distribution and survival status in GSE72094 cohort. (B) Kaplan-Meier survival analysis between high- and low-risk groups in GSE72094 (p < 0.05). (C) ROC curve assessing prognostic performance in GSE72094. (D) Risk score distribution and survival status in GSE31210 cohort. (E) Kaplan-Meier survival analysis between high- and low-risk groups in GSE31210 (p < 0.05). (F) ROC curve evaluating predictive accuracy in GSE31210.
